# Operation for Strictured Hernia

**Published:** 1881-03-20

**Authors:** T. H. Caldwell

**Affiliations:** Rome, Ga.


### OPERATION FOR STRICTURED HERNIA—PATIENT NINETY-TWO YEARS OLD.

BY T. H. CALDWELL, M. D., ROME, GA.

A case came under my observation a few weeks since, which will probably prove interesting to some of the profession, exemplifying, as it does, the remarkable recuperative power sometimes found in persons advanced in years. I was called in to see an old man ninety-two years of age who had an oblique inguinal hernia of twelve years standing.

Two days previous to my visit the gut had fallen. He tried to replace it, as he had done many times previous, but failed. Both my partner, Dr. T. J. Word, and myself, tried by taxis, to reduce the tumor; failing in this, we—using our needle from a hypodermic syringe as a trochar—let off the gas and succeeded in partially reducing it. Upon farther examination we found a commencing hydrocele, caused, probably, from the recent inflammation. We then introduced our needle into the sack and drew off a quantity of bloody serum. The patient was relieved, to a large extent, of his pain, and we left him for the night.

When I called the next day, the gut had refilled with gas and forced itself down once more. I then called in consultation Dr. T. J. Word and Dr. J. B. S Holmes, to consider the advisability of an operation.

Would the age and condition of the patient justify it? As he was, he would surely die within twenty-four hours; but would he survive the operation? We decided to state the case frankly to him and let him decide for himself. This we did. He immediately consented for me to operate. I did so in the usual manner. Upon reaching the seat of the stricture, I discovered the cause of our failure to reduce the tumor after emptying the gut of the gas; there were several very firm adhesions around the ring, and when I broke these up the gut slipped back without any difficulty. The wound healed by first intention. His bowels were moved at the end of six days. The patient has now a good appetite, and is going about as usual.

Taking into consideration the age, the condition of the patient, together with the fact that the intestine had been down for five days previous to the operation, I think it is the most remarkable case of its kind on record.

